# Neuronal injury biomarkers and prognosis in ADNI subjects with normal cognition

**DOI:** 10.1186/2051-5960-2-26

**Published:** 2014-03-06

**Authors:** Jon B Toledo, Michael W Weiner, David A Wolk, Xiao Da, Kewei Chen, Steven E Arnold, William Jagust, Clifford Jack, Eric M Reiman, Christos Davatzikos, Leslie M Shaw, John Q Trojanowski

**Affiliations:** 1 Department of Pathology & Laboratory Medicine, Institute on Aging, Center for Neurodegenerative Disease Research, University of Pennsylvania Perelman School of Medicine, Philadelphia, PA, USA; 2 Center for Imaging of Neurodegenerative Diseases, Department of Radiology, San Francisco VA Medical Center/University of California San Francisco, San Francisco, CA, USA; 3 Department of Neurology, Perelman School of Medicine at the University of Pennsylvania, Philadelphia, PA, USA; 4 Section of Biomedical Image Analysis, Department of Radiology, and Center for Biomedical Image Computing and Analytics, University of Pennsylvania, Philadelphia, PA, USA; 5 Banner Alzheimer's Institute, 901 East Willetta Street, Phoenix, AZ, USA; 6 Department of Psychiatry, Perelman School of Medicine at the University of Pennsylvania, Philadelphia, PA, USA; 7 Helen Wills Neuroscience Institute, University of California, Berkeley, CA, USA; 8 Mayo Clinic College of Medicine, Rochester, MN, USA

**Keywords:** Dementia, Alzheimer’s disease, Magnetic resonance imaging, Cerebrospinal fluid, Amyloid beta, Tau

## Abstract

**Introduction:**

Based on previous studies, a preclinical classification for Alzheimer’s disease (AD) has been proposed. However, 1) specificity of the different neuronal injury (NI) biomarkers has not been studied, 2) subjects with subtle cognitive impairment but normal NI biomarkers (SCINIB) have not been included in the analyses and 3) progression to mild cognitive impairment (MCI) or dementia of the AD type (DAT), referred to here as MCI/DAT, varies between studies. Therefore, we analyzed data from 486 cognitively normal (CN) and 327 DAT subjects in the AD Neuroimaging Initiative (ADNI)-1/GO/2 cohorts.

**Results:**

In the ADNI-1 cohort (median follow-up of 6 years), 6.3% and 17.0% of the CN subjects developed MCI/DAT after 3 and 5 years follow-up, respectively. NI biomarker cutoffs [structural magnetic resonance imaging (MRI), fluorodeoxyglucose positron emission tomography (FDG-PET) and cerebrospinal fluid (CSF) tau] were established in DAT patients and memory composite scores were calculated in CN subjects in a cross-sectional sample (n = 160). In the complete longitudinally followed CN ADNI cohort (n = 326, median follow-up of 2 years), CSF and MRI values predicted an increased conversion to MCI/DAT. Different NI biomarkers showed important disagreements for classifying subjects as abnormal NI [kappa = (−0.05)-(0.33)] and into AD preclinical groups. SCINIB subjects (5.0%) were more prevalent than AD preclinical stage 3 subjects (3.4%) and showed a trend for increased progression to MCI/DAT.

**Conclusions:**

Different NI biomarkers lead to different classifications of ADNI subjects, while structural MRI and CSF tau measures showed the strongest predictive value for progression to MCI/DAT. The newly defined SCINIB category of ADNI subjects is more prevalent than AD preclinical stage individuals.

## Introduction

Alzheimer’s disease (AD) is the most common neurodegenerative disease (ND), characterized and diagnosed by the presence of tau neurofibrillary tangles and amyloid plaques in the central nervous system [1]. Other neurodegenerative and non-degenerative disease pathologies commonly coexist in patients with dementia of the AD type (DAT) and community-dwelling subjects [2-5]. The advent of molecular and neuroimaging AD biomarkers has enabled researchers to better predict the pathologies underlying DAT [6,7] and to formulate research diagnostic criteria [8]. These advances have led to the proposal of a hypothetical AD model [9] for the pathological and biomarker changes to emerge over one or more decades before the onset of dementia or mild cognitive impairment (MCI) [10-12]. It is thought that amyloid deposition precedes cognitive changes by one or more decades and cognitive changes appear when measured amyloid levels approach a plateau. Using this model, a preclinical staging for AD has been proposed based on successive and additive presence of Aβ amyloid deposition (Stage 1), evidence of neuronal injury (NI) biomarkers (Stage 2) and subtle cognitive impairment (Stage 3) all of which precedes MCI and DAT. A separate category for cognitively impaired ADNI subjects with positive NI biomarkers in the absence of Aβ amyloid deposition (suspected non-Alzheimer pathophysiology (sNAP) has also been proposed [13]. Positron emission tomography (PET) imaging with Aβ amyloid ligands and cerebrospinal fluid (CSF) Aβ measurements methods used for estimation of Aβ amyloid deposition are highly correlated [14,15], but for the detection of NI due to AD pathology several other markers are suggested. These include CSF tau, structural magnetic resonance imaging (MRI) and fluorodeoxyglucose PET (FDG-PET). In addition, classification strategies using neuroimaging biomarkers are based on assessments of specific or composite regions of interest (ROI) or pattern analysis methods.

Two studies analyzing different cohorts have described the baseline and longitudinal outcomes of preclinical AD staging with a median follow-up of one and 3.9 years [16,17]. These studies obtained different risk assessments of conversion from CN to MCI or DAT (referred to here as MCI/DAT) and used different sets of NI biomarkers. Although indications are given for the different NI biomarkers [18], no assessment or comparison of the different biomarker modalities and processing has been performed in a single study and this variability might affect the classification of the subjects into the different diagnostic categories. There is another potential and unexplored category of subjects composed of individuals with subtle cognitive impairment with normal neuronal injury biomarkers (SCINIB) independent of the presence or absence of amyloid deposition.

In this study, we 1) compared the agreement of different NI biomarkers and found important differences in prevalence for the different stages of AD, 2) assessed the risk of conversion to DAT in non-demented ADNI subjects that was associated with the different biomarkers to select the best combination of NI biomarkers for the classification of CN subjects, and 3) evaluated the progression of CN subjects to MCI/DAT based on these selected biomarkers.

## Materials and methods

### Participants and neuropsychological testing

**Figure 1:**
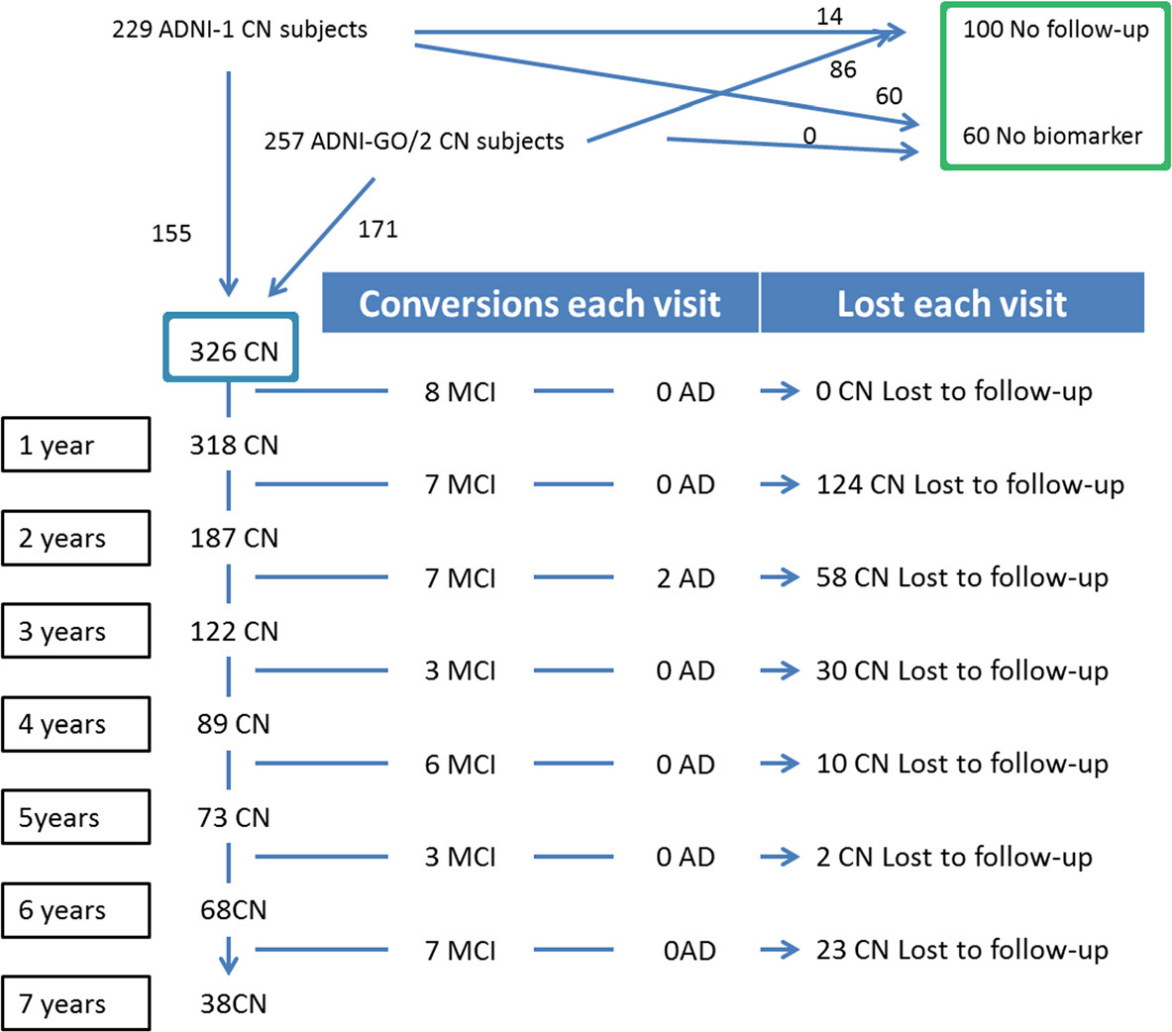
Selection of the cohort and clinical outcomes during follow-up.

Data used in the preparation of this article, was downloaded from the ADNI database November 1^st^ 2013 [19] (http://adni.loni.ucla.edu/ and Additional file 1: supplementary material). Diagnosis of MCI and DAT was established as previously described [20-22] (Additional file 1: Supplementary Material). We included 486ADNI-1/GO/2 CN subjects who were divided into two groups (Figure 1):

a) The first group (Figure 1, blue square) was included in the longitudinal analysis (n = 326), based on a follow-up of at least 1 year and presence of baseline CSF Aβ_1–42_ or FDG PET measurements (Table 1).

b) The second group of CN subjects (Figure 1, green square, Additional file 1:Table S2) was composed of CN subjects without follow-up (n = 100) or without CSF or FDG PET measures (n = 60). These subjects were used to estimate the cutoffs that define subtle cognitive changes for the CN.

**Table 1:** CN ADNI subjects included in the longitudinal study

	**ADNI-1**			**ADNI-GO/2**			**CN stable ADNI-1 vs. ADNI-GO/2**
	**CN stable (n = 120)**	**CN progressors (n = 35)**	**p-value**	**CN stable (n = 163)**	**CN progressors (n = 8)**	**p-value**	**p-value**
Progression to	-	29 MCI	-	-	7 MCI	-	
		6 AD			1 AD		
Age at baseline (years)	74.9 (72.0-78.5)	77.0 (73.0-79.2)	0.37	72.6 (69.4-77.1)	83.0 (80.4-84.8)	0.0001	0.0001
Gender (% male)	52.5%	57.1%	0.49	50.9%	75.0%	0.28	0.89
Education (years)	16.0 (14.0-18.0)	16.0 (13.0-18.0)	0.52	16.0 (15.0-18.5)	17.0 (13.8-18.5)	0.86	0.060
APOE ϵ4 presence	22.5%	31.4%	0.39	29.5%	12.5%	0.44	0.22
ADAS-Cog	9.33 (6.0-12.3)	10.8 (8.6-13.3)	0.047	9.0 (6.0-11)	15.0 (13.5-16.5)	0.0003	0.31
Memory summary score	0.94 (0.66-1.37)	0.71 (0.44-1.01)	0.006	0.94 (0.55-1.22)	0.22 (0.02-0.42)	0.0004	0.16
Executive summary score	0.66 (0.29-1.22)	0.40 (0.03-0.77)	0.039	0.82 (0.40-1.44)	0.23 [(−0.15)-0.47]	0.004	0.091
aHV^1^	812.0 (347.3-1244.5)	586.8 (94.5-1322.6)]	0.25	529.8 (9.0-1085.3)	−226.1 [(−419.3)-(6.7)]	0.007	0.015
SPARE-AD	−1.44 [(−2.15)-(−0.99)]	−1.17 [(−1.74)-(−0.68)]	0.053	−1.32 [(−1.61)-(−1.07)]	−0.90 [(−1.04)-(−0.30)]	0.029	0.019
HCI	5.3 (3.3-7.5)	6.0 (3.9-8.7)	0.20	5.5 (3.5-7.7)	7.2 (3.5-13.6)	0.051	0.29
PC-FDG-PET	1.38 (1.29-1.53)	1.29 (1.23-1.43)	0.022	1.45 (1.33-1.51)	1.31 (1.20-1.36)	0.014	0.54
Aβ_1–42_ (pg/ml)	222.0 (163.5-257.0)	210.0 (144.5-235.0)	0.25	207.7 (158.3-237.3)	147.8 (108.2-205.7)	0.083	0.065
T-tau (pg/ml)	60.0 (47.5-80.8)	71.5 (54.3-95.3)	0.13	56.3 (45.6-81.0)	111.5 (93.7-123.4)	0.032	0.53
P-tau_181_ (pg/ml)	20.0 (16.0-27.5)	22.0 (17.0-31.5)	0.36	30.0 (21.9-43.1)	35.6 (31.0-44.0)	0.25	<0.0001

aHV = adjusted hippocampal volume.

^1^Adjusted for intracranial volume.

Median (1^st^ quartile-3^rd^ quartile).

327 ADNI-1/GO/2 DAT subjects were included to estimate the NI cutoffs for the preclinical AD classification (Additional file 1: Table S2). A summary composite memory measure developed by Crane et al. [23] was used to estimate the presence of subtle cognitive changes.

### CSF biomarker collection and analysis

Aβ_1–42_, t-tau, and p-tau_181_ were measured using the multiplex xMAP Luminex platform (Luminex Corp, Austin, TX) with Innogenetics (INNO-BIA AlzBio3; Ghent, Belgium; for research use–only reagents) immunoassay kit–based reagents (see Additional file 1: supplementary material) [7,24].

### MRI and FDG-PET acquisition and processing

**Figure 2:**
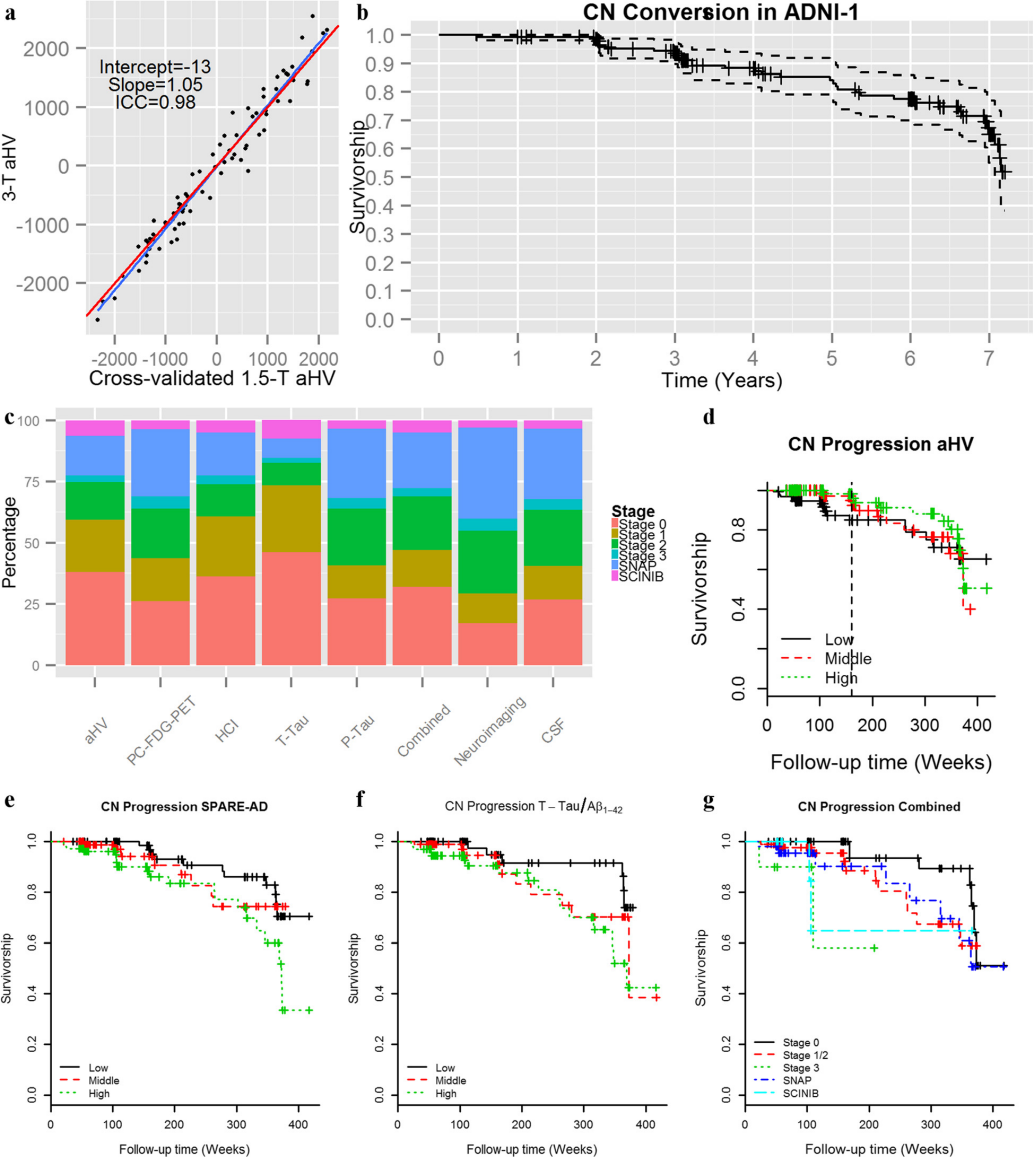
**Validation of aHV transformation, cognitive and biomarker cutoffs and progression of ADNI-1 CN subjects.** Comparisons of cross-validated 1.5-T and corresponding 3-T **(a)**. Progression from CN to MCI/DAT in the ADNI-1 cohort **(b)**. Prevalence of the different CN categories with the use of different neuronal injury biomarkers **(c)**. Conversion of CN subjects to MCI/DAT in adjusted in ADNI-1/GO/2 CN subjects using aHV **(d)** (dotted line represents cutpoint of the heaviside function), SPARE-AD **(e)**, t-tau/Aβ_1–42_ ratio **(f)** and the CN categories defined by the combined NI biomarkers **(g)**.

1.5-T MRI and 3-T non-accelerated sagittal volumetric 3D MPRAGE MRI images were acquired at each performance site for the ADNI 1 and ADNI-GO/2, respectively (http://adni.loni.ucla.edu). Only MRIs which passed the quality control evaluations were included. To estimate hippocampal volumes (HV) measures, cortical grey matter (GM) volumes were processed using Free-surfer software package version 4.4 and 5.1 image processing framework for the 1.5 and 3-T MRI images, respectively (http://surfer.nmr.mgh.harvard.edu/) [25,26]. We estimated in an independent dataset a method to obtain the adjusted HV (aHV; adjusted for intracranial volume (ICV)) for the MRIs (Additional file 1: supplementary material) (Figure 2a). The SPARE-AD (Spatial Pattern of Abnormality for Recognition of Early Alzheimer’s disease) is an index that captures brain atrophy related to AD [27,28]. FDG-PET data were acquired and reconstructed with the use of measured-attenuation correction and the specified reconstruction algorithm for each scanner type according to a standardized protocol (http://adni.loni.ucla.edu/). Images were downloaded and pre-processing using SPM5 by investigators at Banner Alzheimer’s Institute (http://www.fil.ion.ucl.ac.uk/spm). We calculated a pattern based summary score, the hypometabolic convergence index (HCI) [29] and an anatomically defined ROI, the posterior cingulate (PC-FDG-PET with FDG-images using pons as reference region) CMRgl (cerebral metabolic rate for glucose).

### Definition of preclinical AD stages and biomarker and cognitive cutoffs

Presence of Aβ amyloid deposition consistent with AD pathology and T-tau and p-tau_181_ cutoffs were selected based on cutoffs previously validated in a cohort including autopsy confirmed AD subjects [7]. For the MRI and FDG-PET NI biomarkers we did not have any available cutoffs based on a neuropathologically validated sample. We therefore calculated the cutoffs for the remaining NI biomarkers based on values that would give 90% sensitivity for DAT (Additional file 1: Table S3, Additional file 1: Figure S1) [13]. Using this methodology, we could not define a cutoff which was useful for the SPARE-AD score due to its high specificity (only 4.9% of the CN subjects had an abnormal SPARE-AD score). Cutoffs for the memory score indicative of subtle cognitive changes were estimated based on the 10^th^ percentile in the CN subjects not included in the longitudinal analysis [13]. Subjects were categorized as NI presence if any of the two selected biomarkers was abnormal. Subjects were classified into the following categories (Additional file 1: Table S4): 1) Stage 0 [13] (normal Aβ_1–42_, normal NI biomarker and normal cognition), 2) Stage 1 (abnormal Aβ_1–42_, normal NI biomarkers and normal cognition), 3) Stage 2 (abnormal Aβ_1–42_, abnormal NI biomarker and normal cognition), 4) Stage 3 [18] (abnormal Aβ_1–42_, abnormal NI biomarker and abnormal cognition), 5) sNAP [13] (normal Aβ_1–42_ and abnormal NI biomarker) and 6) SCINIB (subtle cognitive impairment with normal NI biomarkers independent of Aβ_1–42_) using the different NI biomarkers.

### Statistical analysis

For the comparison of baseline clinical, biomarker and demographic variables Mann–Whitney U and Kruskall-Wallis tests were applied for the comparison of 2 or 3 groups respectively. For analyses involving an association with longitudinal outcomes, a Box-Cox transformation was applied to non-normally distributed variables. Cutoffs for classification models were selected as described in previous sections. Agreement between the groups defined by the different NI biomarkers was defined using the Cohen’s kappa index. The Cox proportional hazard (PH) model was used to study the progression of CN subjects to MCI/DAT. This model included age, gender education and the presence of APOE ϵ4 allele in addition to the studied biomarker. Quantitative predictors were normalized and standardized in order to be able to compare the effect size of the different NI biomarkers in the PH model. Standardized biomarker values were set so that positive values would indicate abnormal values. The PH assumption was tested analyzing the correlation between the Schoenfeld residuals and survival time. In cases where that the assumption was not meet, a PH with a heaviside function was applied. No correction for multiple comparisons was applied, because all of our NI biomarkers were specified *a priori* based on the recommended NI biomarkers recommended in the preclinical AD criteria [18] and the exploratory nature of our analysis. Statistical significance was set at the *p* < 0.05 level. All statistical tests were two-sided.

## Results

### Description of the cohort

In the total ADNI-1/GO/2 cohort, 43 (8.8%) of the CN subjects converted to MCI, 10 (2.1%) converted to DAT (8 had an MCI diagnosis before the DAT) and 11 (2.3%) died (Table 1 and Additional file 1: Table S2). Of the MCI subjects, 35 (81%) were thought to have a DAT cognitive impairment profile, whereas 8 (19%) were thought to have developed MCI due to other etiologies (Additional file 1: Table S5). All demented patients had a probable DAT diagnosis. In Figure 2b we plot the survival plot for the ADNI-1 CN cohort with a median follow-up of 313 weeks (1^st^ quartile 159 weeks; 3^rd^ quartile: 364 weeks) for comparison with other studies. 86.5% of the ADNI-1 CN subjects had a follow-up of at least 3 years with a progression to MCI/DAT of 6.3%, whereas 56.1% had a follow-up of at least 5 years with a progression to MCI/DAT of 17.0%.

### Comparison of groups based on NI biomarkers and cognitive cutoffs

The different NI biomarkers showed a low agreement, with Cohen’s kappa index values ranging from −0.05 to 0.33 (values below the diagonal in Table 2) and overall agreement between the different NI biomarkers ranged from 45.3% (SPARE-AD and PC-FDG-PET) to 79.0% (SPARE-AD and T-tau). Therefore, the potential use of any single biomarker or combinations of NI biomarkers can lead to important distinctions among the different categories of non-demented ADNI subjects as summarized in Figure 2c.

**Table 2:** Agreement of biomarker measures for NI and subtle cognitive changes

	**aHV**	**SPARE-AD**		**HCI**		**PC-FDG-PET**		**T-tau**		**P-tau**_**181**_	
		**(+)**	**(−)**	**(+)**	**(−)**	**(+)**	**(−)**	**(+)**	**(−)**	**(+)**	**(−)**
**aHV**		66.0%	34.0%	56.4%	43.6%	66.1%	33.9%	60.4%	39.6%	55.75%	44.25%
**SPARE-AD**	0.06			68.5%	31.5%	50.7%	49.3%	79.0%	21.0%	45.3%	54.7%
**HCI**	0.05	0.05				64.5%	45.5%	63.6%	36.4%	46.0%	54.0%
**PC-FDG-PET**	0.33	0.03		0.10				50.8%	49.2%	51.0%	49.0%
**T-tau**	0.06	0.04		0.11		0.07				63.6%	36.4%
**P-tau**_**181**_	0.15	0.0		−0.01		−0.05		0.31			

Numbers below the diagonal represent Cohen’s kappa index. Numbers above the diagonal represent the percentage of subjects that were classified the same way by the pair of NI biomarkers (+) and the percentage of cases that were classified differently by the pair of NI biomarkers (−).

### Clinical progression based on the different NI biomarkers and cognitive measures

Due to the absence of any specific recommendations regarding the use of different combinations of NI biomarkers to classify the CN subjects, we tested the associated risk of progression of CN subjects to MCI/DAT based on the different NI biomarkers in the Cox PH models (Table 3). Only the MRI and the t-tau/Aβ_1–42_ values were associated with a higher risk of progression to MCI/DAT (Figure 2d-f) while lower baseline memory measures were the strongest predictors. Finally, we also selected for further analysis a biomarker from each modality showing the strongest association with progression, i.e.t-tau for the CSF and aHV for the neuroimaging, and called this model the combined NI model.

**Table 3:** **Association between NI, tau/Aβ**_**1–42**_**ratios and cognitive scores in CN subject with conversion to MCI/DAT**

**Neuronal injury marker**	**Hazard ratio**	**95% confidence interval**	**p-value**
aHV: <=160 weeks	3.11	1.84-5.25	<0.0001
aHV: >160 weeks^1^	0.92	0.56-1.53	0.76
SPARE-AD	1.46	1.12-1.92	0.006
HCI	1.26	0.84-1.88	0.27
PC-FDG-PET	1.37	0.92-2.03	0.12
T-tau/Aβ_1–42_	1.60	1.09-2.36	0.016
P-tau/Aβ_1–42_	1.49	0.52-2.26	0.065
Memory summary score	2.46	1.69-3.56	<0.0001

^1^For aHV a heaviside function with a time cutpoint of 160 was selected based on the distribution of the Schoenfeld residuals indicating that after 3 years group did not differ in risk.

Cox hazards models were adjusted for age, education, gender and APOE ϵ4 presence. Biomarker values were normalized and standardized for comparison.

### Clinical progression based on the preclinical AD stages using different combinations of NI biomarkers and clinical measures

Of the 326 ADNI-1/GO/2 subjects with longitudinal follow-up, 238 had measurements for the selected NI biomarkers. Five out of the twelve SCINIB subjects had abnormal Aβ_1–42_. The association of the different categories with progression to MCI/DAT is summarized in and Table 4 (Figure 2g). Stage 3 was associated with progression to MCI/DAT and the SCINIB category showed a trend. Additional file 1: Table S6 lists the results obtained using neuroimaging-only or CSF-only NI biomarkers. When subjects were categorized using only CSF NI biomarker the Stage 3, we found that subjects in the sNAP or SCINIB category were associated with progression to MCI/DAT. In none of the models did stage 1 and 2 show an association with faster progression.

**Table 4:** Association between preclinical AD stages and conversion to MCI/DAT

**Neuronal injury marker**	**Percentage of subjects in each category**	**Total number of subjects (Subjects who progressed)**	**Hazard ratio (95% CI)**	**p-value**
Combined-NI	Stage 0: 31.9%	76 (7)	Ref.	Ref.
	Stage 1: 15.1%	36 (5)	2.6 (0.8-8.6)	0.12
	Stage 2: 21.8%	52 (6)	1.8 (0.5-6.3)	0.34
	Stage 3: 3.4%	8 (2)	11.3 (1.9-66.9)	0.0072
	SNAP: 22.7%	54 (8)	2.4 (0.8-6.9)	0.12
	SCINIB: 5.0%	12 (2)	4.9 (0.8-29.1)	0.078

Cox hazards models were adjusted for age, gender and APOE ϵ4 presence.

## Discussion

Our study describes for the first time the unexplored variability of NI biomarkers among CN subjects, and we found that CSF tau and structural MRI measures, either aHV or SPARE-AD, were the strongest predictors of conversion to MCI/DAT from among a very comprehensive set of NI biomarkers. Selecting the best biomarkers, we classified the CN subjects and included the SCINIB category in our analyses since they had not been analysed in previous study, and we showed a higher prevalence of the SCINIB category than the AD preclinical stage 3. While only the AD preclinical stage was associated with increased progression to MCI/DAT, the SCINIB category showed a trend for progression which could become significant with longer follow up of these subjects.

Two previous studies have described the distribution of the AD preclinical stages and the progression of CN to MCI/DAT [16,17] and a third study has described the neuropsychological changes, but not the diagnostic changes associated with the preclinical stages of AD [30]. In the Washington University (WU) study, with a median follow-up of 3.9 years, the 5-year progression from CN to a clinical dementia rating of at least 0.5 deemed to be due to AD was 10% [17]. On the other hand, the Mayo Clinic (MC) population-based study showed the same progression rate, namely 10%, but with a follow-up of a single year. In our study, the conversion from CN to MCI/DAT was 6.3% at 3 years of follow-up and 17.0% at 5 years of follow-up in the ADNI-1 cohort (median follow-up of five years). Neither the ADNI nor the WU cohorts are population-based studies like the MC cohort and comparisons should be performed to assess baseline differences that explain these findings. In addition a third study described longitudinal memory and executive decline in AD preclinical stages 1 and 2 but not in the sNAP category, although conversion to MCI/DAT was not studied [30].

In our study we included a wide range of standardized AD biomarker measurements that are used as measures of NI in the preclinical AD criteria [18]. In addition, for the MRI and FDG-PET we included two types of measures, i.e. regions of interest and machine learning methods. Similarly, two NI measures were available for the CSF, namely t-tau and p-tau_181_. The performed analyses showed that all the NI measures, even those within the same modality showed an important disagreement for the classification of subjects according to the consistent absence or presence of NI biomarkers (Table 2 and Figure 2c). This is not surprising due to the fact that NI biomarkers track changes in different stages of the disease and at a different rate [9]. For example, in this study aHV was only associated with faster progression in the first years. The measures that showed the highest agreement were CSF t-tau and p-tau_181_, which showed a high correlation as well as PC-FDG-PET and aHV, as described previously [31,32]. In addition, biomarkers with high sensitivity and specificity, like the SPARE-AD, cannot be used to categorize subjects using the previous approaches [13] due to the small overlap between CN and DAT subjects and therefore cutoffs based on the longitudinal outcomes might be needed for biomarkers with a high accuracy. Many NI biomarkers might not be disease specific. This is, for example, the case of MRI HV and medial temporal lobe measures that can be affected by different ND and show additive effect from ND [5,33,34]. This also can be the case of FDG-PET measures. Nevertheless, p-tau_181_, which would be expected to be the most specific NI biomarker, was the one that was associated with the highest prevalence of sNAP cases. Interestingly, a recent study reported that in some cases incident amyloid positivity is preceded by NI positivity [35]. These results underscore the importance of standardized studies which include different NI measures in order to assess the implications of using different biomarkers and how this can affect comparability of different studies.

The WU study used the presence of either abnormal t-tau or p-tau_181_ as NI biomarkers and the MC study used the presence of either abnormal FDG–PET or HCV. None of the studies assessed the impact of using a wider panel of different NI measures. From a diagnostic point of view, specific criteria are needed to define the different preclinical AD stages and studies should assess the different sources of variability for the different NI biomarkers as well as the specificity that each one offers.

Whereas from a research perspective it might be important to examine and compare in the same study different types of biomarkers this is not case in clinical scenarios that require cost effective and reproducible measures linked to clinical outcomes. Here, we studied several biomarkers in the ADNI cohort and found that structural MRI and CSF t-tau were the best predictors for conversion to MCI/DAT, and therefor they were used for the combined model. This is in agreement with previous studies that have shown that either brain atrophy [36,37] or CSF biomarkers [30,38,39] are associated with an increased risk of progression of CN subjects to MCI/DAT. Finally, a recent study in a small subset of ADNI patients has shown that a combination of biomarkers can predict the conversion from CN subjects to MCI/DAT [40] and therefore biomarkers combinations might be able to predict the appearance of cognitive symptoms in subjects at risk with higher accuracy than the preclinical stages and reflecting the different underlying pathologies in subjects with cognitive impairment [5].

SCINIB is a new category outside the AD hypothetical model that includes subjects with subtle cognitive changes who were not previously identified by the array of NI biomarkers used in AD studies. This category was more prevalent in the ADNI cohort than the stage 3 group using the combined NI model. The SCINIB group was composed of a mixture of subjects with normal and abnormal CSF Aβ_1–42_ values and this group showed a trend for increased conversion to MCI/DAT. Previous studies have not included this group in their main analyses, because investigators have focused on validating the preclinical AD stages or subjects with NI measures. However, this might lead to the impression that the preclinical staging explains most of the conversion of CN subjects to MCI/DAT. It is not surprising that the SCINIB group might be associated with clinical progression because it is defined by neuropsychological measures that are also in part used to establish the clinical diagnosis (but this would also apply to the preclinical AD stage 3 groups). This finding underscores the importance of not excluding SCINIB subjects from studies and characterizing them longitudinally in order to understand their longitudinal prognosis and potential biomarkers that identify these subjects.

## Conclusion

We confirm that there is increased progression for the AD preclinical stage 3 and probably SCINIB, but there is a high classification variability regarding the AD preclinical, sNAP and SCINIB categories based on the selection of the NI biomarkers that may reflect different aspects of disease. Therefore specific and standardized criteria are needed to be able to apply a reproducible and robust classification strategy and new approaches for the definition of cutoffs will be needed for biomarker with a high accuracy. In addition, a large percentage of subjects with baseline subtle memory changes fell into the SCINIB category, which needs further study to characterize its longitudinal outcome and the underlying pathological changes.

## Competing interests

Dr. Weiner reports stock/stock options from Elan, Synarc, travel expenses from Novartis, Tohoku University, Fundacio Ace, Travel eDreams, MCI Group, NSAS, Danone Trading, ANT Congress, NeuroVigil, CHRU-Hopital Roger Salengro, Siemens, AstraZeneca, Geneva University Hospitals, Lilly, University of California, San Diego–ADNI, Paris University, Institut Catala de Neurociencies Aplicades, University of New Mexico School of Medicine, Ipsen, Clinical Trials on Alzheimer’s Disease, Pfizer, AD PD meeting, Paul Sabatier University, board membership for Lilly, Araclon, Institut Catala de Neurociencies Aplicades, Gulf War Veterans Illnesses Advisory Committee, VACO, Biogen Idec, Pfizer, consultancy from AstraZeneca, Araclon, Medivation/Pfizer, Ipsen, TauRx Therapeutics, Bayer Healthcare, Biogen Idec, ExonHit Therapeutics, Servier, Synarc, Pfizer, Janssen, honoraria from NeuroVigil, Insitut Catala de Neurociencies Aplicades, PMDA/Japanese Ministry of Health, Labour, and Welfare, Tohoku University, commercial research support from Merck, Avid; government research support, DOD, VA, outside the submitted work. Dr. Shaw serves as consultant for Janssen AI R & D Janssen AI R & D and Lilly, outside the submitted work. Dr. Jagust has served as consultant for Genentech, Synarc, Siemens, F. Hoffman La Roche, Tau Rx, and Janssen Alzheimer Immunotherapy, outside the submitted work. Dr. Arnold reports grants from NIH, the American Health Assistance Foundation and the Marian S Ware Alzheimer’s Program, several pharmaceutical companies, other from Universities, pharmaceutical companies and advisory/speaking honoraria from Universities, pharmaceutical companies and law firms. Dr. Jack, Reiman, Chen, Wolk, Davatzikos, Da and Toledo have nothing to disclose.

## Authors’ contributions

All authors read and approved the final manuscript, contributed to interpretation of the data and critical review of the manuscript and study concept. XD and CD processed and analyzed the MRI data. KC and EMR processed and analyzed the FDG-PET data. JBT drafted the manuscript and performed the statistical analyses. JQT drafted the manuscript.

## Supplementary Material

Additional file 1: Table S1 ADNI 1 criteria for recruitment of CN and DAT subjects. **Table S2.** ADNI-1 and ADNI-GO/2 DAT patients included to derive cutoff values and CN subjects without longitudinal follow-up or lack of CSF or FDG-PET measurements. **Table S3.** Biomarker and clinical cutoffs with 90% DAT sensitivity and corresponding specificities obtained in CN not included in longitudinal analysis and cutoffs based on 10^th^ percentile in CN not included in longitudinal analysis. **Table S4.** Criteria for classifying ADNI subjects into the different CN, prodromal DAT and clinically manifest DAT categories described in this study. **Table S5.** Clinical diagnoses of MCI subjects whose impairment was not attributed to AD. **Table S6.** Association between preclinical AD stages and conversion to MCI/DAT. Cox hazards models were adjusted for age, gender and APOE ϵ4 presence. Figure S1. Neuronal injury and memory cutoffs. aHV (a), SPARE-AD (b), HCI (c), FDG-PET ROI score (d) and memory composite score (e) values in CN and DAT subjects in the samples of subjects used for the estimation of cutoffs. Dashed line represents the selected cutoff. Click here for file

## References

[B1] Montine TJ Phelps CH Beach TG Bigio EH Cairns NJ Dickson DW Duyckaerts C Frosch MP Masliah E Mirra SS Nelson PT Schneider JA Thal DR Trojanowski JQ Vinters HV Hyman BT National Institute on Aging-Alzheimer's Association guidelines for the neuropathologic assessment of Alzheimer's disease: a practical approach Acta neuropathologica 2012 2 1 11 10.1007/s00401-011-0910-3 22101365 PMC3268003

[B2] Toledo JB Brettschneider J Grossman M Arnold SE Hu WT Xie SX Lee VM Shaw LM Trojanowski JQ CSF biomarkers cutoffs: the importance of coincident neuropathological diseases Acta Neuropathol 2012 2 23 35 10.1007/s00401-012-0983-7 22526019 PMC3551449

[B3] Toledo JB Arnold SE Raible K Brettschneider J Xie SX Grossman M Monsell SE Kukull WA Trojanowski JQ Contribution of cerebrovascular disease in autopsy confirmed neurodegenerative disease cases in the National Alzheimer's Coordinating Centre Brain: J neurol 2013 2 2697 2706 10.1093/brain/awt188 PMC3858112 23842566

[B4] Schneider JA Arvanitakis Z Bang W Bennett DA Mixed brain pathologies account for most dementia cases in community-dwelling older persons Neurology 2007 2 2197 2204 10.1212/01.wnl.0000271090.28148.24 17568013

[B5] Toledo J Cairns N Da X Chen K Carter D Fleisher A Householder E Ayutyanont N Roontiva A Bauer R Eisen P Shaw LM Davatzikos C Weiner MW Reiman EM Morris JC Trojanowski JQ Clinical and multimodal biomarker correlates of ADNI neuropathological findings Acta Neuropathologica Communications 2013 2 65 10.1186/2051-5960-1-65 24252435 PMC3893373

[B6] McMillan CT Irwin DJ Avants BB Powers J Cook PA Toledo JB McCarty Wood E Van Deerlin VM Lee VM Trojanowski JQ Grossman M White matter imaging helps dissociate tau from TDP-43 in frontotemporal lobar degeneration J Neurol Neurosurg Psychiatry 2013 2 949 955 10.1136/jnnp-2012-304418 23475817 PMC3737288

[B7] Shaw LM Vanderstichele H Knapik-Czajka M Clark CM Aisen PS Petersen RC Blennow K Soares H Simon A Lewczuk P Dean R Siemers E Potter W Lee VM Trojanowski JQ Cerebrospinal fluid biomarker signature in Alzheimer's disease neuroimaging initiative subjects Annals of neurology 2009 2 403 413 10.1002/ana.21610 19296504 PMC2696350

[B8] McKhann GM Knopman DS Chertkow H Hyman BT Jack CR Jr Kawas CH Klunk WE Koroshetz WJ Manly JJ Mayeux R Mohs RC Morris JC Rossor MN Scheltens P Carrillo MC Thies B Weintraub S Phelps CH The diagnosis of dementia due to Alzheimer's disease: recommendations from the National Institute on Aging-Alzheimer's Association workgroups on diagnostic guidelines for Alzheimer's disease Alzheimer's & dementia: the journal of the Alzheimer's Association 2011 2 263 269 10.1016/j.jalz.2011.03.005 PMC3312024 21514250

[B9] Jack CR Jr Knopman DS Jagust WJ Petersen RC Weiner MW Aisen PS Shaw LM Vemuri P Wiste HJ Weigand SD Lesnick TG Pankratz VS Donohue MC Trojanowski JQ Tracking pathophysiological processes in Alzheimer's disease: an updated hypothetical model of dynamic biomarkers Lancet neurology 2013 2 207 216 10.1016/S1474-4422(12)70291-0 23332364 PMC3622225

[B10] Villemagne VL Burnham S Bourgeat P Brown B Ellis KA Salvado O Szoeke C Macaulay SL Martins R Maruff P Ames D Rowe CC Masters CL Amyloid beta deposition, neurodegeneration, and cognitive decline in sporadic Alzheimer's disease: a prospective cohort study Lancet neurology 2013 2 357 367 10.1016/S1474-4422(13)70044-9 23477989

[B11] Toledo JB Xie SX Trojanowski JQ Shaw LM Longitudinal change in CSF Tau and Abeta biomarkers for up to 48 months in ADNI Acta Neuropathol 2013 2 659 670 10.1007/s00401-013-1151-4 23812320 PMC3875373

[B12] Jack CR Jr Wiste HJ Lesnick TG Weigand SD Knopman DS Vemuri P Pankratz VS Senjem ML Gunter JL Mielke MM Lowe VJ Boeve BF Petersen RC Brain beta-amyloid load approaches a plateau Neurology 2013 2 890 896 10.1212/WNL.0b013e3182840bbe 23446680 PMC3653215

[B13] Jack CR Jr Knopman DS Weigand SD Wiste HJ Vemuri P Lowe V Kantarci K Gunter JL Senjem ML Ivnik RJ Roberts RO Rocca WA Boeve BF Petersen RC An operational approach to National Institute on Aging-Alzheimer's Association criteria for preclinical Alzheimer disease Annals of neurology 2012 2 765 775 10.1002/ana.22628 22488240 PMC3586223

[B14] Toledo JB Vanderstichele H Figurski M Aisen PS Petersen RC Weiner MW Jack CR Jr Jagust W Decarli C Toga AW Toledo E Xie SX Lee VM Trojanowski JQ Shaw LM Factors affecting Abeta plasma levels and their utility as biomarkers in ADNI Acta neuropathologica 2011 2 401 413 10.1007/s00401-011-0861-8 21805181 PMC3299300

[B15] Landau SM Lu M Joshi AD Pontecorvo M Mintun MA Trojanowski JQ Shaw LM Jagust WJ Comparing positron emission tomography imaging and cerebrospinal fluid measurements of beta-amyloid Annals of neurology 2013 2 826 836 10.1002/ana.23908 23536396 PMC3748164

[B16] Knopman DS Jack CR Jr Wiste HJ Weigand SD Vemuri P Lowe V Kantarci K Gunter JL Senjem ML Ivnik RJ Roberts RO Boeve BF Petersen RC Short-term clinical outcomes for stages of NIA-AA preclinical Alzheimer disease Neurology 2012 2 1576 1582 10.1212/WNL.0b013e3182563bbe 22551733 PMC3348848

[B17] Vos SJ Xiong C Visser PJ Jasielec MS Hassenstab J Grant EA Cairns NJ Morris JC Holtzman DM Fagan AM Preclinical Alzheimer's disease and its outcome: a longitudinal cohort study Lancet Neurol 2013 2 957 965 10.1016/S1474-4422(13)70194-7 24012374 PMC3904678

[B18] Sperling RA Aisen PS Beckett LA Bennett DA Craft S Fagan AM Iwatsubo T Jack CR Jr Kaye J Montine TJ Park DC Reiman EM Rowe CC Siemers E Stern Y Yaffe K Carrillo MC Thies B Morrison-Bogorad M Wagster MV Phelps CH Toward defining the preclinical stages of Alzheimer's disease: recommendations from the National Institute on Aging-Alzheimer's Association workgroups on diagnostic guidelines for Alzheimer's disease Alzheimer's & dementia: the journal of the Alzheimer's Association 2011 2 280 292 10.1016/j.jalz.2011.03.003 PMC3220946 21514248

[B19] Weiner MW Veitch DP Aisen PS Beckett LA Cairns NJ Green RC Harvey D Jack CR Jagust W Liu E Morris JC Petersen RC Saykin AJ Schmidt ME Shaw L Shen L Siuciak JA Soares H Toga AW Trojanowski JQ The Alzheimer's Disease Neuroimaging Initiative: a review of papers published since its inception Alzheimer's & dementia: the journal of the Alzheimer's Association 2013 2 e111 e194 10.1016/j.jalz.2013.05.1769 PMC4108198 23932184

[B20] Petersen RC Smith GE Waring SC Ivnik RJ Tangalos EG Kokmen E Mild cognitive impairment: clinical characterization and outcome Arch Neurol 1999 2 303 308 10.1001/archneur.56.3.303 10190820

[B21] Petersen RC Aisen PS Beckett LA Donohue MC Gamst AC Harvey DJ Jack CR Jr Jagust WJ Shaw LM Toga AW Trojanowski JQ Weiner MW Alzheimer's Disease Neuroimaging Initiative (ADNI): clinical characterization Neurology 2010 2 201 209 10.1212/WNL.0b013e3181cb3e25 20042704 PMC2809036

[B22] McKhann G Drachman D Folstein M Katzman R Price D Stadlan EM Clinical diagnosis of Alzheimer's disease: report of the NINCDS-ADRDA Work Group under the auspices of Department of Health and Human Services Task Force on Alzheimer's Disease Neurology 1984 2 939 944 10.1212/WNL.34.7.939 6610841

[B23] Crane PK Carle A Gibbons LE Insel P Mackin RS Gross A Jones RN Mukherjee S Curtis SM Harvey D Weiner M Mungas D Development and assessment of a composite score for memory in the Alzheimer's Disease Neuroimaging Initiative (ADNI) Brain imaging and behavior 2012 2 502 516 10.1007/s11682-012-9186-z 22782295 PMC3806057

[B24] Shaw LM Vanderstichele H Knapik-Czajka M Figurski M Coart E Blennow K Soares H Simon AJ Lewczuk P Dean RA Siemers E Potter W Lee VM Trojanowski JQ Qualification of the analytical and clinical performance of CSF biomarker analyses in ADNI Acta neuropathologica 2011 2 597 609 10.1007/s00401-011-0808-0 21311900 PMC3175107

[B25] Reuter M Rosas HD Fischl B Highly accurate inverse consistent registration: a robust approach NeuroImage 2010 2 1181 1196 10.1016/j.neuroimage.2010.07.020 20637289 PMC2946852

[B26] Reuter M Schmansky NJ Rosas HD Fischl B Within-subject template estimation for unbiased longitudinal image analysis NeuroImage 2012 2 1402 1418 10.1016/j.neuroimage.2012.02.084 22430496 PMC3389460

[B27] Fan Y Shen D Gur RC Gur RE Davatzikos C COMPARE: classification of morphological patterns using adaptive regional elements IEEE Trans Med Imaging 2007 2 93 105 17243588 10.1109/TMI.2006.886812

[B28] Toledo JB Da X Bhatt P Wolk DA Arnold SE Shaw LM Trojanowski JQ Davatzikos C Relationship between plasma analytes and SPARE-AD defined brain atrophy patterns in ADNI PloS one 2013 2 e55531 10.1371/journal.pone.0055531 23408997 PMC3568142

[B29] Chen K Ayutyanont N Langbaum JB Fleisher AS Reschke C Lee W Liu X Bandy D Alexander GE Thompson PM Shaw L Trojanowski JQ Jack CR Jr Landau SM Foster NL Harvey DJ Weiner MW Koeppe RA Jagust WJ Reiman EM Characterizing Alzheimer's disease using a hypometabolic convergence index NeuroImage 2011 2 52 60 10.1016/j.neuroimage.2011.01.049 21276856 PMC3066300

[B30] van Harten AC Smits LL Teunissen CE Visser PJ Koene T Blankenstein MA Scheltens P van der Flier WM Preclinical AD predicts decline in memory and executive functions in subjective complaints Neurology 2013 2 1409 1416 10.1212/WNL.0b013e3182a8418b 24049134

[B31] Greicius MD Srivastava G Reiss AL Menon V Default-mode network activity distinguishes Alzheimer's disease from healthy aging: evidence from functional MRI Proc Natl Acad Sci USA 2004 2 4637 4642 10.1073/pnas.0308627101 15070770 PMC384799

[B32] Fouquet M Desgranges B Landeau B Duchesnay E Mezenge F de la Sayette V Viader F Baron JC Eustache F Chetelat G Longitudinal brain metabolic changes from amnestic mild cognitive impairment to Alzheimer's disease Brain : J Nneurol 2009 2 2067 2058 10.1093/brain/awp132 PMC2936690 19477964

[B33] Wilson RS Yu L Trojanowski JQ Chen EY Boyle PA Bennett DA Schneider JA TDP-43 Pathology, cognitive decline, and dementia in old age JAMA neurology 2013 2 1418 10.1001/jamaneurol.2013.3961 24080705 PMC3830649

[B34] Nelson PT Smith CD Abner EL Wilfred BJ Wang WX Neltner JH Baker M Fardo DW Kryscio RJ Scheff SW Jicha GA Jellinger KA Van Eldik LJ Schmitt FA Hippocampal sclerosis of aging, a prevalent and high-morbidity brain disease Acta neuropathologica 2013 2 161 177 10.1007/s00401-013-1154-1 23864344 PMC3889169

[B35] Jack CR Jr Wiste HJ Weigand SD Knopman DS Lowe V Vemuri P Mielke MM Jones DT Senjem ML Gunter JL Gregg BE Pankratz VS Petersen RC Amyloid-first and neurodegeneration-first profiles characterize incident amyloid PET positivity Neurology 2013 2 1732 1740 10.1212/01.wnl.0000435556.21319.e4 24132377 PMC3821718

[B36] Rusinek H De Santi S Frid D Tsui WH Tarshish CY Convit A de Leon MJ Regional brain atrophy rate predicts future cognitive decline: 6-year longitudinal MR imaging study of normal aging Radiology 2003 2 691 696 10.1148/radiol.2293021299 14657306

[B37] Driscoll I Davatzikos C An Y Wu X Shen D Kraut M Resnick SM Longitudinal pattern of regional brain volume change differentiates normal aging from MCI Neurology 2009 2 1913 1906 10.1212/WNL.0b013e3181a82634 PMC2690968 19487648

[B38] Fagan AM Roe CM Xiong C Mintun MA Morris JC Holtzman DM Cerebrospinal fluid tau/beta-amyloid (42) ratio as a prediction of cognitive decline in nondemented older adults Archives of neurology 2007 2 343 349 10.1001/archneur.64.3.noc60123 17210801

[B39] Li G Sokal I Quinn JF Leverenz JB Brodey M Schellenberg GD Kaye JA Raskind MA Zhang J Peskind ER Montine TJ CSF tau/Abeta42 ratio for increased risk of mild cognitive impairment: a follow-up study Neurology 2007 2 631 639 10.1212/01.wnl.0000267428.62582.aa 17698783

[B40] Rizk-Jackson A Insel P Petersen R Aisen P Jack C Weiner M Early Indications of Future Cognitive Decline: Stable versus Declining Controls PloS one 2013 2 e74062 10.1371/journal.pone.0074062 24040166 PMC3767625

