# Supplementary material for: Neuronal injury biomarkers and prognosis in ADNI subjects with normal cognition
Source: Acta Neuropathol Commun. 2014 Mar 6;2:26. doi: 10.1186/2051-5960-2-26 (PMC4008258; doi:10.1186/2051-5960-2-26)
Supplement: Additional file 1: Table S1 — ADNI 1 criteria for recruitment of CN and DAT subjects. Table S2. ADNI-1 and ADNI-GO/2 DAT patients included to derive cutoff values and CN subjects without longitudinal follow-up or lack of CSF or FDG-PET measurements. Table S3. Biomarker and clinical cutoffs with 90% DAT sensitivity and corresponding specificities obtained in CN not included in longitudinal analysis and cutoffs based on 10th percentile in CN not included in longitudinal analysis. Table S4. Criteria for classifying ADNI subjects into the different CN, prodromal DAT and clinically manifest DAT categories described in this study. Table S5. Clinical diagnoses of MCI subjects whose impairment was not attributed to AD. Table S6. Association between preclinical AD stages and conversion to MCI/DAT. Cox hazards models were adjusted for age, gender and APOE ϵ4 presence. Figure S1. Neuronal injury and memory cutoffs. aHV (a), SPARE-AD (b), HCI (c), FDG-PET ROI score (d) and memory composite score (e) values in CN and DAT subjects in the samples of subjects used for the estimation of cutoffs. Dashed line represents the selected cutoff. [file 2051-5960-2-26-S1.docx]

**Neuronal Injury Biomarker Variability and Prognosis in ADNI Subjects with Normal Cognition**

Jon B. Toledo, Michael W. Weiner, David A. Wolk, Xiao Da, Kewei Chen, Steven E Arnold, William Jagust, Clifford Jack, Eric M. Reiman, Christos Davatzikos, Leslie M. Shaw, and John Q. Trojanowski

**Methods**

ADNI description and goals

The ADNI was launched in 2003 by the National Institute on Aging (NIA), the National Institute of Biomedical Imaging and Bioengineering (NIBIB), the Food and Drug Administration (FDA), private pharmaceutical companies and non-profit organizations, as 5-year public-private partnership. The primary goal of ADNI has been to test whether serial MRI [[1](#_ENREF_1), [2](#_ENREF_2)], PET [[3](#_ENREF_3)], other biological markers [[4](#_ENREF_4)], and clinical and neuropsychological assessment [[5](#_ENREF_5)] can be combined to measure the progression of MCI and early AD. Determination of sensitive and specific markers of very early AD progression is intended to aid researchers and clinicians to develop new treatments and monitor their effectiveness, as well as lessen the time and cost of clinical trials.

The Principal Investigator of this initiative is Michael W. Weiner, MD, VA Medical Center and University of California – San Francisco. ADNI is the result of efforts of many co-investigators from a broad range of academic institutions and private corporations, and subjects have been recruited from over 50 sites across the U.S. and Canada. The initial goal of ADNI was to recruit 800 subjects but ADNI has been followed by ADNI-GO and ADNI-2. To date these three protocols have recruited over 1500 adults, ages 55 to 90, to participate in the research, consisting of cognitively normal older individuals, people with early or late MCI, and people with early AD. The follow up duration of each group is specified in the protocols for ADNI-1, ADNI-2 and ADNI-GO. Subjects originally recruited for ADNI-1 and ADNI-GO had the option to be followed in ADNI-2. For up-to-date information, see www.adni-info.org.

Recruitment inclusion and exclusion criteria for ADNI 1

Inclusion criteria were as follows: 1) Hachinski Ischemic Score ≤4; 2) Permitted medications stable for 4 weeks prior to screening; 3) Geriatric Depression Scale score < 6; 4) visual and auditory acuity adequate for neuropsychological testing; good general health with no diseases precluding enrollment; 5) 6 grades of education or work history equivalent; 6) Ability to speak English or Spanish fluently; 7) A study partner with 10 hours per week of contact either in person or on the telephone and who could accompany the participant to the clinical visits.

Criteria for the different diagnostic groups are summarized in supplementary table 1. Groups were age matched. CN subjects could not have any significant cognitive impairment or impaired activities of daily living. Clinical diagnosed Alzheimer’s disease patients (cAD), i.e. those with DAT had a mild degree of dementia and meet the National Institute of Neurological and Communicative Disorders and Stroke–Alzheimer’s Disease and Related Disorders Association criteria for probable DAT [[6](#_ENREF_6)], whereas MCI should not meet these criteria and they have largely intact general cognition and functional performance with only mild reductions in their neuropsychology test scores as described (cite Petersen or an ADNI publication for this such as the 2013 Weiner et al review of ADNI.

*CSF Biomarker collection and analysis*

CSF was collected into polypropylene collection tubes or syringes provided to each site, then transferred into polypropylene transfer tubes without any centrifugation step followed by freezing on dry ice within 1 hour after collection, and shipped overnight to the ADNI Biomarker Core laboratory at the University of Pennsylvania Medical Center on dry ice. Aliquots (0.5 ml) were prepared from these samples after thawing (1 hour) at room temperature and gentle mixing. The aliquots were stored in bar code–labeled polypropylene vials at -80°C. Fresh, never before thawed 0.5 mL aliquots for each subject’s set of longitudinal time points were analyzed on the same 96 well plate in the same analytical run for this study in order to minimize run to run and reagent kit lot sources of variation. The capture monoclonal antibodies used were 4D7A3 for Aβ_1-42_, AT120 for t-tau and AT270 for p-tau_181_. The analyte-specific detector antibodies were HT7, for tau, and 3D6, for the N-terminus of Aβ [[7](#_ENREF_7)]. Within run coefficient of variation (%CV) for duplicate samples ranged from 2.5-5.9% for Aβ_1-42_, 2.2-6.3% for t-tau and 2.0-6.5% for p-tau_181_ and the inter-run %CV for CSF pool samples ranged from 5.1-14% for Aβ_1-42_, 2.7 -11.2% for t-tau and 3.3-11.5% for p-tau_181_. Further information on the procedures and standard operating procedures can be found in previous publications [[4](#_ENREF_4), [7](#_ENREF_7)] and online (<http://www.adni-info.org/>). Upennbiomk4 and upennbiomk5 measurements have been anchored as detailed in the whitepaper available in LONI (<http://www.loni.usc.edu>).

Evaluation of 1.5-T MRI processed with Freesurfer v4.4 and 3-T MRI processed with Freesurfer v5.1

As described in the manuscript MRI images ADNI-1 subjects were acquired using 1.5-T MRI fields and processed using Free-surfer software package version 4.4, whereas for ADNI-GO/2 1.5-T MRI fields and Free-surfer software package version 5.1 were used. To test if these differences would affect the estimated aHV, we cross-validated the aHV using 1.5-T and 3-T measurements transformations that were obtained during the same visit for a subset of 273 ADNI-1 subjects. We selected 70% of the subjects (n=191) to calculate the transformation formula that we then applied to the remaining 82 subjects. Five-fold cross-validation was applied in the training sample to select the linear transformations.

We calculated the 1.5-T aHV in the training set and then transformed it to match the 3-T aHV values in the training set. Then we used these transformations on the test set (**Figure 2a**). The obtained intercept, slope, coefficients of determination (R^2^) and intraclass correlation coefficient were -12.7, 1.05, 0.96 and 0.98, respectively.

**Supplementary table 1**. ADNI 1 criteria for recruitment of CN and DAT subjects.

|  | CN | DAT |
| --- | --- | --- |
| Memory complaints | Absent | Present |
| MMSE | 24-30 | 20-26 |
| CDR | 0 | 0.5-1.0 |
| Delayed recall Logical Memory II subscale of WMSR | 16 YoEd: ≥9  8–15 YoEd: ≥5  0-7 YoEd: ≥3 | 16 YoEd: ≤8  8–15 YoEd: ≤4  0-7 YoEd: ≤2 |

CDR: Clinical Dementia Rating; MMSE: Mini-Mental State Examination; YoEd : years of education. ^1^Mandatory requirement of the memory box score being 0.5 or greater.

**Supplementary table 2. ADNI-1 and ADNI-GO/2 DAT patients included to derive cutoff values and CN subjects without longitudinal follow-up or lack of CSF or FDG-PET measurements.**

|  | ADNI-1 | | ADNI-GO/2 | |
| --- | --- | --- | --- | --- |
|  | CN  (n=74) | DAT  (n=191) | CN  (n=86) | DAT  (n=136) |
| Diagnosis | 7 CN to MCI  3 CN to AD | - | - | - |
| Age at baseline (years) | 74.9 (72.5-77.7) | 75.7 (70.8-80.7) | 70.7 (67.4-76.7) | 75.4 (70.9-79.8) |
| Gender (% male) | 48.6% | 52.4% | 34.8% | 59.6% |
| Education (years) | 16.0 (15.0-18.0) | 15.0 (12.0-16.0) | 18.0 (16.0-18.0) | 16.0 (14.0-18.0) |
| e-ANART | 7.0 (3.0-12.0) | - | 8.0 (4.0-13.0) | - |
| APOE ε4 presence (%) | 31.1% | 66.0% | 33.3% | 70.9% |
| ADAS-Cog | 9.0 (5.8-12.2) | 28.7 (23.7-34.0) | 8.0 (5.3-12.0) | 31.0 (25.0-36.0) |
| Memory summary score | 1.0 (0.6-1.4) | -0.82 [(-0.5)-(-1.2)] | 0.9 (0.6-1.2) | -0.6 [(-0.8)-(-0.4)] |
| Executive summary score | 0.8 (0.5-1.2) | -0.9 [(-0.4)-(-1.5)] | 0.5 (0.2-1.0) | -0.8 [(-1.4)-(-0.3)] |
| aHV^1^ | 957.2 (170.5-1400.1) | -889.8 [(-1358.7)-(-233.2)] | 840.6 (235.4-1273.1) | -1229.8 [(-1649.5)-(-626.4)] |
| SPARE-AD | -1.3 [(-1.7)-(-1.0)] | 1.3 (0.8-1.7) | -1.3 [(-1.6)-(-1.1)] | 0.9 (0.4-1.3) |
| HCI | 5.3 (3.8-6.0) | 12.5 (9.6-17.4) | 3.9 (2.7-5.9) | 13.1 (8.8-16.1) |
| PC-FDG-PET | 1.41 (1.3-1.5) | 1.2 (1.1-1.3) | 1.4 (1.3-1.5) | 1.2 (1.1-1.3) |

aHV= adjusted hippocampal volume.

^1^ Adjusted for intracranial volume.

Median (1^st^ quartile-3^rd^ quartile).

**Supplementary table 3. Biomarker and clinical cutoffs with 90% DAT sensitivity and corresponding specificities obtained in CN not included in longitudinal analysis and cutoffs based on 10^th^ percentile in CN not included in longitudinal analysis**.

|  | Biomarker | Measure | Value | Specificity |
| --- | --- | --- | --- | --- |
| Cognitive based on DAT subjects | MRI | Hippocampal volume | 327.0 | 70.8% |
|  |  | SPARE-AD^1^ | 0.25 | 95.1% |
|  | FDG-PET | HCI | 6.97 | 85.7% |
|  |  | PC-FDG-PET | 1.40 | 57.1% |
| Cognitive based on CN subjects | Cognitive measures | Memory | 0.31 | - |
|  |  | Executive | -0.16 | - |

^1^ Only available in ADNI-1 subjects.

**Supplementary table 4. Criteria for classifying ADNI subjects into the different CN, prodromal DAT and clinically manifest DAT categories described in this study.**

|  | Abnormal Aβ | Abnormal Neuronal Injury | Abnormal Cognitive Changes |
| --- | --- | --- | --- |
| Stage 0 | - | - | - |
| Stage 1 | + | - | - |
| Stage 2 | + | + | - |
| Stage 3 | + | + | + |
| sNAP | - | + | ± |
| SCINIB | ± | - | + |

+: Abnormal; -: Normal; ±: Normal or abnormal.

**Supplementary table 5. Clinical diagnoses of MCI subjects whose impairment was not attributed to AD.**

| Diagnosis | ADNI-1 | ADNI-GO/2 |
| --- | --- | --- |
| Depression | 3 | - |
| Possible depression and medication | 1 | - |
| Stress related due to new physical diagnosis | 1 | - |
| Vascular disease | 1 | - |
| Major depression and vascular dementia | 1 | - |
| Uncertain | 1 | - |

**Supplementary table 6.** **Association between preclinical AD stages and conversion to MCI/DAT.** Cox hazards models were adjusted for age, gender and APOE ε4 presence.

| Neuronal Injury Marker | Percentage of subjects in each category | Total number of subjects (Subjects who progressed) | Hazard ratio (95%CI) | p-value |
| --- | --- | --- | --- | --- |
| Neuroimaging-based NI^1^ | Stage 0: 17.1%  Stage 1: 12.1%  Stage 2: 25.6%  Stage 3: 5.0%  SNAP: 37.2%  SCINIB: 3.0% | 34 (1)  24 (2)  51 (6)  10 (3)  74 (6)  6 (1) | -  Ref.  1.4 (0.2-8.6)  6.6 (0.7-59.8)  0.7 (0.1-3.7)  3.2 (0.3-38.5) | -  Ref.  0.73  0.093  0.66  0.35 |
| CSF-based NI | Stage 0: 26.8%  Stage 1: 13.6%  Stage 2: 23.0%  Stage 3: 4.3%  SNAP: 28.8%  SCINIB: 3.5% | 69 (7)  35 (6)  59 (6)  11 (3)  74 (8)  8 (3) | Ref.  2.9 (1.0-12.7)  2.6 (0.8-10.2)  14.1 (3.3-76.8)  3.1 (1.2-11.3)  5.9 (1.3-31.0) | Ref.  0.084  0.13  0.00075  0.041  0.024 |

^1^None of the Stage 0 CN subjects progressed to MCI/DAT and therefore stage 1 was selected as reference category (model did not converge for stage 0).

**Supplementary figure 1. Neuronal injury and memory cutoffs.** aHV (a), SPARE-AD (b), HCI (c), FDG-PET ROI score (d) and memory composite score (e) values in CN and DAT subjects in the samples of subjects used for the estimation of cutoffs. Dashed line represents the selected cutoff.


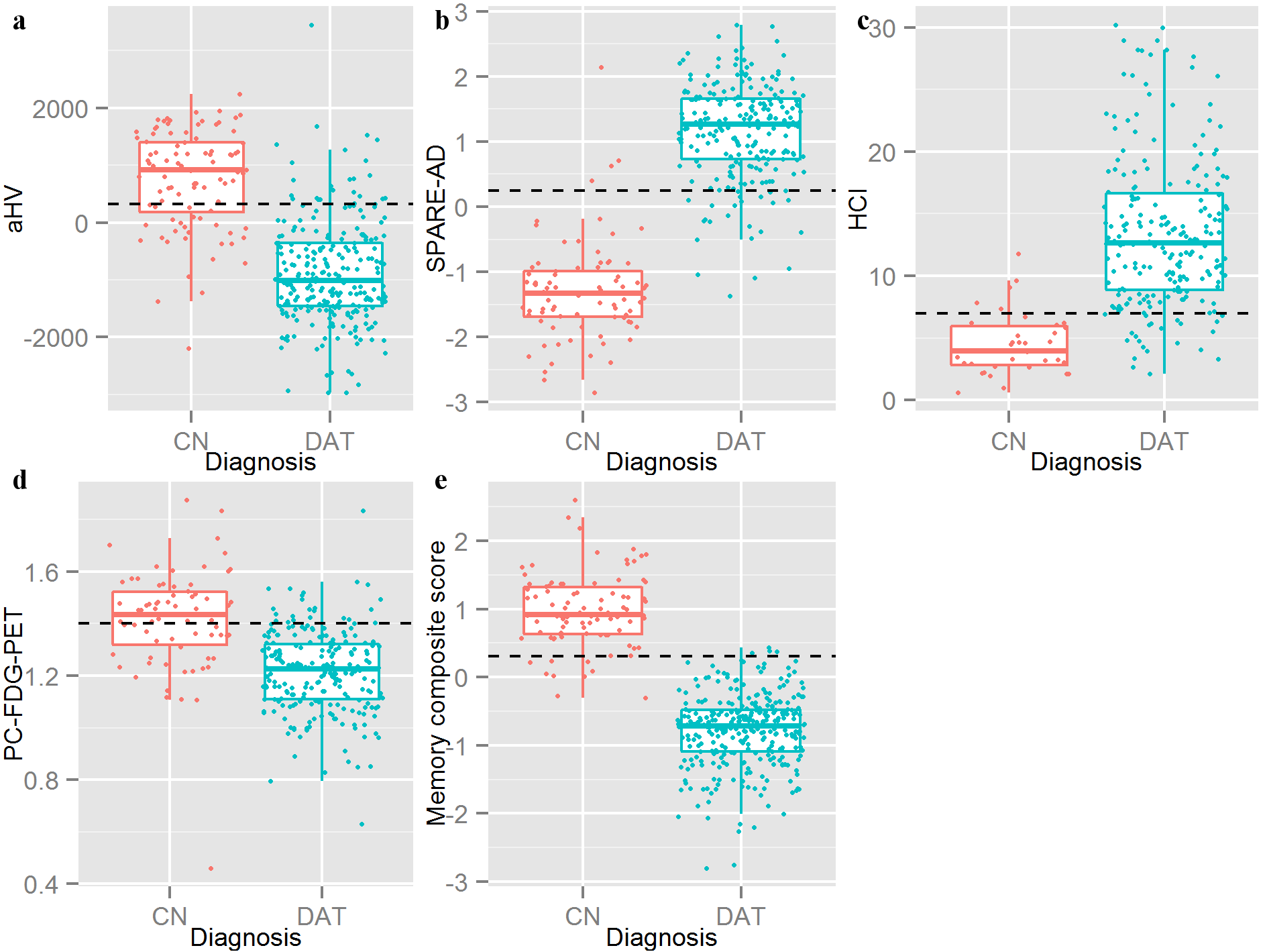


**References**

1. Jack CR, Jr., Bernstein MA, Fox NC, Thompson P, Alexander G, Harvey D, Borowski B, Britson PJ, J LW, Ward C, et al: **The Alzheimer's Disease Neuroimaging Initiative (ADNI): MRI methods.** *Journal of magnetic resonance imaging : JMRI* 2008, **27:**685-691.

2. Weiner MW, Veitch DP, Aisen PS, Beckett LA, Cairns NJ, Green RC, Harvey D, Jack CR, Jagust W, Liu E, et al: **The Alzheimer's Disease Neuroimaging Initiative: a review of papers published since its inception.** *Alzheimer's & dementia : the journal of the Alzheimer's Association* 2012, **8:**S1-68.

3. Jagust WJ, Bandy D, Chen K, Foster NL, Landau SM, Mathis CA, Price JC, Reiman EM, Skovronsky D, Koeppe RA: **The Alzheimer's Disease Neuroimaging Initiative positron emission tomography core.** *Alzheimer's & dementia : the journal of the Alzheimer's Association* 2010, **6:**221-229.

4. Shaw LM, Vanderstichele H, Knapik-Czajka M, Clark CM, Aisen PS, Petersen RC, Blennow K, Soares H, Simon A, Lewczuk P, et al: **Cerebrospinal fluid biomarker signature in Alzheimer's disease neuroimaging initiative subjects.** *Annals of neurology* 2009, **65:**403-413.

5. Petersen RC, Aisen PS, Beckett LA, Donohue MC, Gamst AC, Harvey DJ, Jack CR, Jr., Jagust WJ, Shaw LM, Toga AW, et al: **Alzheimer's Disease Neuroimaging Initiative (ADNI): clinical characterization.** *Neurology* 2010, **74:**201-209.

6. McKhann G, Drachman D, Folstein M, Katzman R, Price D, Stadlan EM: **Clinical diagnosis of Alzheimer's disease: report of the NINCDS-ADRDA Work Group under the auspices of Department of Health and Human Services Task Force on Alzheimer's Disease.** *Neurology* 1984, **34:**939-944.

7. Shaw LM, Vanderstichele H, Knapik-Czajka M, Figurski M, Coart E, Blennow K, Soares H, Simon AJ, Lewczuk P, Dean RA, et al: **Qualification of the analytical and clinical performance of CSF biomarker analyses in ADNI.** *Acta neuropathologica* 2011, **121:**597-609.
